# Surgical Approach to the Adrenal Glands

**DOI:** 10.17925/EE.2015.11.02.98

**Published:** 2015-08-19

**Authors:** David Scott-Coombes

**Affiliations:** Consultant in Endocrine Surgery, Department of Endocrine Surgery, University Hospital of Wales, Cardiff, UK

**Keywords:** Adrenal, adrenalectomy, laparoscopic, phaeochromocytoma, outcomes

## Abstract

Any surgeon treating a patient with adrenal disease should be a member of a multi-disciplinary team involving dedicated specialists, including an endocrinologist, anaesthetist, radiologist, intensivist and geneticist. In an era of epidemic numbers of adrenal incidentalomas, great care must be taken to determine not only the endocrine diagnosis, but also the benefits (if any) of adrenal surgery. Finally, the surgeon must be competent in both minimally invasive and gross resectional surgical techniques and know when to adopt these two very different surgical approaches.

The five surgical tenets of endocrine surgery were set out in the 1980s:^1^

- Confirm the diagnosis;
- Render the patient safe;
- Consider localisation studies;
- Decide if surgery is indicated; and
- Decide which surgical approach to adopt.

Confirmation of the diagnosis is based exclusively on biochemical tests and the surgeon should be able to interpret the results almost as well as an endocrinologist. For functioning tumours, the multi-disciplinary team (MDT) should be content that the patient is as stable as possible prior to surgery. For phaeochromocytoma, in the UK, phenoxybenzamine is the commonest choice of drug to deliver alpha-adrenergic receptor blockade with minimal beta-adrenergic blockade prescribed later if a reflex tachycardia develops. Typical UK practice commences pharmacological blockade as an outpatient and then as an inpatient the dose is titrated upwards in the immediate pre-operative period until a satisfactory postural drop in blood pressure has been achieved. Patients with Cushing’s syndrome may require medication to control excess cortisol production prior to surgery. Hypertension and hypokalaemia are relatively easily controlled in most patients with Conn’s syndrome.^2^

Cross-sectional imaging is undertaken in all patients with adrenal disease. It will lateralise the pathology in patients with Cushing’s syndrome and exclude the possibility of a paraganglioma in patients with a biochemical diagnosis of phaeochromocytoma. Cross-sectional imaging will also indicate the size of the tumour and provide information about the likelihood of malignancy. MIBG is reserved in patients with a phaeochromocytoma when either metastasis is suspected or no primary tumour is identified. For patients with a biochemical diagnosis of Conn’s syndrome, adrenal venous sampling should be undertaken to confirm the laterality of the disease.^3^

There are two broad surgical approaches: endoscopic or laparotomy By the year 2000 laparoscopic adrenalectomy became the gold standard approach.^4^ As with cholecystectomy, there has not been a randomised trial, but case series have demonstrated several advantages of laparoscopic over open surgery. These include less pain, shorter inpatient stay and quicker return to work with equivalent morbidity and mortality rates.^5^ Endoscopic approaches mirror the open approaches that include retroperitoneal and transperitoneal routes. The advantages of a retroperitoneal approach include the avoidance of repositioning a patient undergoing a bilateral adrenalectomy and the lack of a need to mobilise either the liver or the spleen/pancreas.^6^ For huge tumours the open access can be widened using either an extended lateral incision, or a transthoracic approach (either via a midline sternotomy or a lateral approach). The decision about open or ‘keyhole’ surgery is mainly influenced by two factors: tumour size and likelihood of malignancy.

Depending upon the experience of the surgeon, a laparoscopic approach is limited to tumours up to about 10 cm in diameter. Of course the tumour needs to be extracted and large tumours will require a larger incision! There is an option to perform a hand-assisted procedure (popular in renal transplant surgery) where a ‘hand-port’ that admits one (surgeon’s) hand into the peritoneal cavity is used to both dissect and facilitate tumour extraction. When a malignancy is suspected, laparotomy is the favoured approach. Adrenocortical cancer is highly malignant and the best option for cure is to remove the tumour with a clear margin (R_0_ resection).^7^ Malignant tumours are typically large and have a propensity to local invasion. The surgeon must be prepared to resect neighbouring tissues that can include kidney, pancreas, spleen and liver. These rare tumours should be treated in a few centres where a team of surgeons with appropriate skills can be tailored to the individual patient/tumour.

## Operating Influencing Factors

Factors that influence the operative approach are related to the patient, the tumour or the surgeon.

### Patient Factors

Previous abdominal surgery might cause adhesions and impede a transperitoneal laparoscopic route. This is a major advantage of the retroperitoneal approach, but it is limited to tumours up to 50 mm in diameter. Morbid obesity is not a contraindication to a laparoscopic approach and benefits the larger patient more so than a smaller patient.

### Tumour Factors

As stated, tumour size restricts the options for both retroperitoneal and transperitoneal approaches. Aside from phaeochromocytoma, a larger size is associated with a greater risk of malignancy. While laparoscopic surgery has been shown to be safe in medium-term follow-up for tumours diagnosed as malignant post-operatively,^8^ when malignancy is suspected pre-operatively laparotomy is the appropriate approach (see above). Tumours that are sited in awkward areas, especially around the renal hilum are less likely to be successfully removed with a laparoscopic approach.

### Surgeon Factors

Adrenal surgeons must be able to perform endoscopic surgery. However, retroperitoneal surgery is not widely practised and is only available in a few centres. Currently too many surgeons in the UK are undertaking adrenal surgery. The median number of adrenals excised by a surgeon in England is one per year. The relationship between surgeon volume and patient outcome is well established in adrenal surgery^9^ and guidelines recommending not only MDTs but also a minimum volume of cases per annum would be welcomed. All surgeons undertaking adrenal surgery should submit their outcomes to the UK national endocrine surgery registry.

Good peri-operative care is crucial. This is especially so in Cushing’s, where the disease has often resulted in severe osteoporosis. Physical transfer of patients and patient positioning on the operating table must be undertaken with the greatest care. As the liver is typically fatty and platelet function is compromised, the surgeon must strive to be as gentle as possible during dissection and liver retraction and meticulous with haemostasis.^10^ A peri-operative steroid replacement regimen should be planned and prescribed.

During removal of a phaeochromocytoma there should be a constant dialogue between surgeon and anaesthetist particularly prior to clamping the main adrenal vein to avoid hypotension. The traditional practice of recovering patients with phaeochromocytoma in Critical Care has diminished for most patients, mainly due to better pre- and peri-operative care.

## Conclusion

After a clear biochemical diagnosis and multi-disciplinary decision to perform adrenalectomy, laparoscopic surgery is the gold standard approach. This results in short hospital stay and speedy recovery. The decision to perform open surgery is mainly influenced by the tumour size and the risk of malignancy – for which open surgery is the only option. Peri-operative care is vital to obtain good outcomes with minimal complications.
